# The Medical Profession and the Public Well-Being

**Published:** 1921-08-27

**Authors:** 


### The Hospital

The Workers' Newspaper of Administrative Medicine and Institutional Life, Administration, National Insurance and Health.

No. 1838, Vol. LXX. SATURDAY, AUGUST 27, 1921. PRICE TWOPENCE

### THE MEDICAL PROFESSION AND THE PUBLIC WELL-BEING.

A good many medical economists have been exercising their minds recently on the prospects of medicine as a career for young men—and for young women too—and on the probable developments of the future as they affect the interests of those already in, or about to become members of, the medical profession in this country. It is pointed out that just now there is a "boom" in medical study; that the students registering during the past two or three years so vastly exceed in numbers the records of the past that such factors as war wastage will soon be negligible as affecting the numbers of those entitled to call themselves registered practitioners; that medical schools and colleges are so crowded as to make it very difficult to provide for the accommodation and training of those who seek instruction; that women are rushing into the profession in such proportions as not merely to compete seriously with their male colleagues, but as also to find little elbow-room among those branches of professional work to which women have established special and undeniable claims.

With such pessimistic forebodings ringing in their ears, parents, guardians, and ambitious young people of both sexes may well pause over the choice of medicine as a career. And it is right that everyone who proposes to adopt this profession should think very seriously, and more than once, before taking the final decision. That is not to say—far from it—that the warnings and croakings of every pessimist are to be taken at their face value. As will be explained in more detail presently, the long view of medicine, difficult though it be for the individual to be sure he sees it aright, discloses to the writer, at all events, no discouraging prospect. Of hard work, even of drudgery, the intending medical man and woman must not be afraid, both before and after qualification; of fortune, materially speaking, they must not pitch any high expectation; of strenuous competition they must not prove apprehensive. But when all this is admitted it needs but little vision to perceive how many new fields of varied activity are opening up to medical men and women, and how much wider a choice of activity awaits them to-day than was vouchsafed to their predecessors of past generations.

It is a truism, a platitude, perhaps (though truisms and platitudes are necessary in educational numbers of medical journals, if nowhere else), to illustrate this theorem by mentioning the careers that are afforded by the developments of the Public Health Services, such as school medical inspection, tuberculosis work, infant-welfare work, venereal clinics, and so forth. Large numbers of men, and especially large percentages of women, entering the medical profession find in these salaried appointments, with pension rights and definite hours "on duty," work congenial to their tastes and requirements. Yet in "private practice" also conditions are changing, and scope for individual development is greater than it has ever been before. It is private practice not merely as such, but as an integral part of the Health Service and sanitary progress of the kingdom as a whole, that engulfs the majority of those who enter the medical profession; and it is the private practitioner, many contend, who is capable of being and ought to be—will be—the greatest single asset of public health and national well-being that is anywhere to be found. The new specialties constantly springing up—such as radiology, bacteriology, and vaccine therapy, to say nothing of the subdivisions of surgery and medicine—provide the individual with ample choice if specialisation appeals to him. But it is the general practitioner to whom the greatest opportunities are granted, and it is he whose outlook has been most altered by the trend of legislation and of current social evolution.

Many pieces of evidence could be quoted in support of this generalisation, some of greater, some of less significance. The National Health Insurance Acts, though they may not have fulfilled all the rosy expectations formed at their inception, have at least recognised the private practitioner as the foundation-stone of this great effort to improve the national health and its standards. Not every panel practitioner, it must be admitted, is worthy of his hire; but it is something that a decent standard of hire is assured to him, and improvement in the general average level of his skill and conscientiousness is most certainly occurring. Just at the moment the projects which some idealists have propounded for a general State Medical Service are out of favour; whether they will be taken seriously in the near future no one can say, but at least it is safe to prophesy that they have no chance of fruition or of acceptance unless they are based upon the efficient general practitioner and his encouragement.

Moreover, it is but a few weeks since a correspondent pleaded in our own columns for the general practitioner to ally himself definitely with preventive, as well as curative medicine, by giving lectures to his circle of patients on the maintenance of their health before, rather than during, actual illness; and many voices have urged lately that the public at large should go to a doctor once a month or once a quarter in order that the earliest beginnings of disease shall not escape observation, as so often happens at present. Yet again, the "team work" of the famous Mayo brothers at Rochester, in Minnesota, if it has not yet been challenged by anything equally elaborate on this side, is at least making men think seriously: one hears of "teams" of doctors taking up panel work in association, each one studying some specialty in addition to doing the ordinary routine work of general practice.

Let us take it for granted, therefore, that in the future, as in the past, and more than in the past, the general practitioner is to be an indispensable part of the organisation of the medical profession. What of the competition likely to result from the overcrowding which is being foretold as likely to result in four and five and six years' time? Well, in the first place, there is an old saying, trite enough but truer than youth usually realises, that there is always room at the top. The man and woman of bright intelligence, reasonable health, and real energy and industry need not bother about the fierceness of the competition they are likely to meet with; all forms of professional life involve competition, and the best need not fear that they will fail to win through. For weaklings, of course, there is little room in the hurly-burly of practice; the salaried forms of State or municipal service attract many whose health hardly admits of such a strain. Since much of the tendency of modern research is in the direction of prevention of disease, it will be incumbent on the general practitioner of the future to know more of this matter than satisfied the last generation. Fortunately, the rising band of practitioners who have served in the war have had ample object-lessons of the rôle which such measures as preventive inoculation can play in the suppression of infective diseases, and may be trusted to keep an open mind regarding such discoveries and developments as the next few years may bring forth. For the rest, we must trust to those who are more directly in control of medical education to see to it that those about to qualify realise to the full their part in the constant struggle against disease. *Imprimis*, the General Medical Council, which exercises statutory powers of control over examining bodies; then the examining bodies themselves; and lastly the teachers at the medical schools and the authors of text-books for students, on whom lie very onerous responsibilities for the wise direction of the studies of those to whose hands the next generation of the English people will entrust its lives and its health.

It is, on the whole, with optimism and confidence rather than with forebodings that we regard the future of the medical profession in this country. Material rewards on the scale of those obtainable in commerce, or even in some of the sister professions, its members never did, and probably never will, command. But if they can retain and deserve the confidence of their fellow-citizens, as we hope and believe they will on the whole, and play a worthy part in the social progress of the British nation, theirs is no mean lot; and the mere fact, if fact it turn out to be, that unusual numbers of men and women are seeking to join their ranks does not cause us to swerve from that view, for we believe that there is work, albeit of various kinds, for them to do.

